# Analysis of molecular characteristics of CAMP-negative *Streptococcus agalactiae* strains

**DOI:** 10.3389/fmicb.2023.1189093

**Published:** 2023-05-24

**Authors:** Jie Zhou, Li Zhang, Yang Zhang, Hui Liu, Kangli Xu, Baohu Zhang, Tianyuan Feng, Shucai Yang

**Affiliations:** ^1^Department of Clinical Laboratory, Pingshan General Hospital, Southern Medical University (Pingshan District People’s Hospital of Shenzhen), Shenzhen, Guangdong, China; ^2^Department of Pharmacy, Pingshan General Hospital, Southern Medical University (Pingshan District People’s Hospital of Shenzhen), Shenzhen, Guangdong, China

**Keywords:** CAMP, MLST, *Streptococcus agalactiae*, CFB, GBS

## Abstract

**Background:**

*Streptococcus agalactiae* can produce CAMP factor, which can promote the β-hemolysin activity of *Staphylococcus aureus*, forming an arrow-shaped hemolysis enhancement zone at the intersection of the two bacterial species on a blood agar plate. This characteristic feature of *Streptococcus agalactiae* has led to the widespread use of the CAMP test as an identification method.

**Methods:**

Vaginal/rectal swabs, collected from women at 35–37  weeks of pregnancy, were first inoculated into a selective enrichment broth media, then subcultured onto GBS chromogenic agar and 5% sheep blood agar sequentially. The VITEK-2 automatic identification system and MALDI-TOF MS were initially employed for identification, followed by the CAMP test. CAMP-negative strains underwent 16S rDNA and *cfb* gene sequence analysis, as well as bacterial multilocus sequence typing.

**Results:**

A total of 190 strains were isolated, with 15 identified as CAMP-negative. Further 16S rDNA gene sequence analysis confirmed that all 15 strains were *Streptococcus agalactiae*. The MLST typing assay revealed that these 15 strains were of the ST862 type. The *cfb* gene was amplified and electrophoresed, but no specific fragments were found, indicating that these strains lack the CAMP factor due to *cfb* gene deletion. Antibiotic susceptibility tests demonstrated no resistance to penicillin, ampicillin, vancomycin and linezolid among the GBS strains. However, there are significant differences in resistance rates to tetracycline.

**Conclusion:**

This study found that 7.9% of GBS strains isolated from the vagina/rectum of pregnant women were CAMP-negative, suggesting that the CAMP test method or primers targeting the *cfb* gene should not be used as the sole presumptive test for GBS identification.

## 1. Introduction

*Streptococcus agalactiae*, also known as group B *Streptococcus* (GBS), is a Gram-positive coccus commonly found in the gastrointestinal and genitourinary tracts. Research indicates that approximately 11 to 35% of pregnant women are colonized by GBS in the vagina or rectum. (Russell et al., 2017; Bogiel et al., 2021). GBS is the primary pathogen responsible for neonatal infections, with mother-to-child transmission being the predominant mode of infection. Roughly 50% of GBS-colonized pregnant women transmit the bacteria to their newborns. In the absence of intrapartum antibiotic prophylaxis (IAP), 1% ~ 2% of newborns may develop sepsis, meningitis, pneumonia, or other serious complications, potentially leading to neonatal death or neurological sequelae (Puopolo et al., 2019; Geteneh et al., 2020). Since the early 1990s in the United States, GBS screening and IAP have effectively reduced the incidence of neonatal infections caused by GBS (Schrag et al., 2002; Nanduri et al., 2019).

CAMP factor is the primary virulence factor of GBS. The CAMP reaction was initially characterized as the synergistic lysis of sheep red blood cells by *Staphylococcus aureus* sphingomyelinase and CAMP factor (Christie et al., 1944). For an extended period, the CAMP phenomenon has served as a crucial basis for the laboratory diagnosis of GBS (Jin et al., 2018). Concurrently, the gene encoding CAMP factor, the *cfb* gene, has been utilized as the target of PCR assays for GBS diagnosis (Carrillo-Avila et al., 2018).

Although the CAMP test is a vital identification method for GBS, instances of GBS with negative CAMP tests have been reported occasionally (Guo et al., 2019). In this study, we employed GBS chromogenic agar plates to screen GBS from perinatal pregnant women, which demonstrated higher sensitivity compared to blood agar plates. We identified 15 strains of CAMP-negative GBS. Our laboratory has conducted preliminary molecular characteristics analyses of these strains, and the findings are presented as follows:

## 2. Materials and methods

### 2.1. Strain collection and identification

A total of 1,391 vaginal/rectal swabs were collected from pregnant women at 35–37 weeks of gestation between April 2020 and March 2021 at Pingshan General Hospital of Southern Medical University. To enhance detection rates, the vaginal/rectal swabs were inoculated into a selective enrichment broth media (Jiangmen Kailin Trading Co., Ltd., China) and incubated for 18–24 h at 35–37°C in 5% CO_2_ conditions. Subsequently, the samples were subcultured onto GBS chromogenic agar plates (Zhengzhou Antu Biological Engineering Co., Ltd., China) for approximately 24 h. The purple colonies were then selected and cultivated on 5% sheep blood agar (Guangzhou Dijing Microbial Science and Technology, China) for another 24 h. Suspected isolates were initially identified using the VITEK-2 automatic identification system (BioMérieux, France) and confirmed by MALDI-TOF MS (BioMérieux, France).

### 2.2. CAMP test

The CAMP reactions of isolates identified as *Streptococcus agalactiae* were assessed on 5% sheep blood agar (Guangzhou Dijing Microbial Science and Technology, China) following conventional methods (Guo et al., 2019), *Streptococcus agalactiae* (ATCC13813) was employed as a positive control, *Enterococcus faecalis* (ATCC29212) as a negative control, and *Staphylococcus aureus* (ATCC25923) for the production of β-hemolysin.

### 2.3. 16S rDNA and cfb gene sequencing

Genomic DNA was isolated and purified using the bacterial genomic extraction kit DP302 (TIANGEN Biotech, Beijing, China). The 16S rDNA gene and *cfb* gene were amplified using Applied Biosystems 7,500 (Thermo Fisher, Foster City, USA) with primers as reported (Hongoh et al., 2003; Cezarino et al., 2008). Additionally, we designed another set of primers targeting the *cfb* gene for further verification (Forward: 5′-TGGTAGTCGTGTAGAAGCCTTA-3′; Reverse: 5′-TCCAACAGCATGTGTGATTGC-3′). All amplified fragments were analyzed by agarose gel electrophoresis and sent to Shanghai Personalbio Technology for sequencing. The obtained sequences were blasted in NCBI database. *Streptococcus agalactiae* (ATCC13813) was used as a positive control in the assay.

### 2.4. Multilocus sequence typing

Seven housekeeping genes of GBS (*adhP*, *pheS*, *atr*, *glnA*, *sdhA*, *glcK* and *tkt*) were amplified separately with primers reported (Jones et al., 2003). The amplified products were analyzed by agarose gel electrophoresis and sent to Shanghai Personalbio Technology for sequencing. The obtained sequences were submitted to the MLST analysis website^1^ to obtain allele numbers and STs.

### 2.5. Antibiotic susceptibility testing

VITEK-2 susceptibility testing, including penicillin, ampicillin, clindamycin, erythromycin, levofloxacin, tetracycline, linezolid and vancomycin, was conducted following the manufacturer’s instructions using the AST-GP67 card. The results obtained after a maximum of 15 h of incubation were analyzed and interpreted by AES 7.01 software. *Staphylococcus aureus* (ATCC29213) and *Enterococcus faecalis* (ATCC29212) were used as quality control strains to ensure the credibility of the results. The D-zone test with erythromycin and clindamycin (OXOID, United Kingdom) placed at 12 mm (edge to edge) distance was performed on 5% sheep blood MH agar (Guangzhou Dijing Microbial Science and Technology, China) and incubated for 20–24 h at 37°C.

### 2.6. Statistical analysis

The data were reported as numbers (percentages) and compared using the chi-square test. *p* values <0.05 were considered statistically significant. All data were analyzed using the statistical software SPSS 19.0.

## 3. Results

### 3.1. Multilocus sequence typing

**Figure 1:**
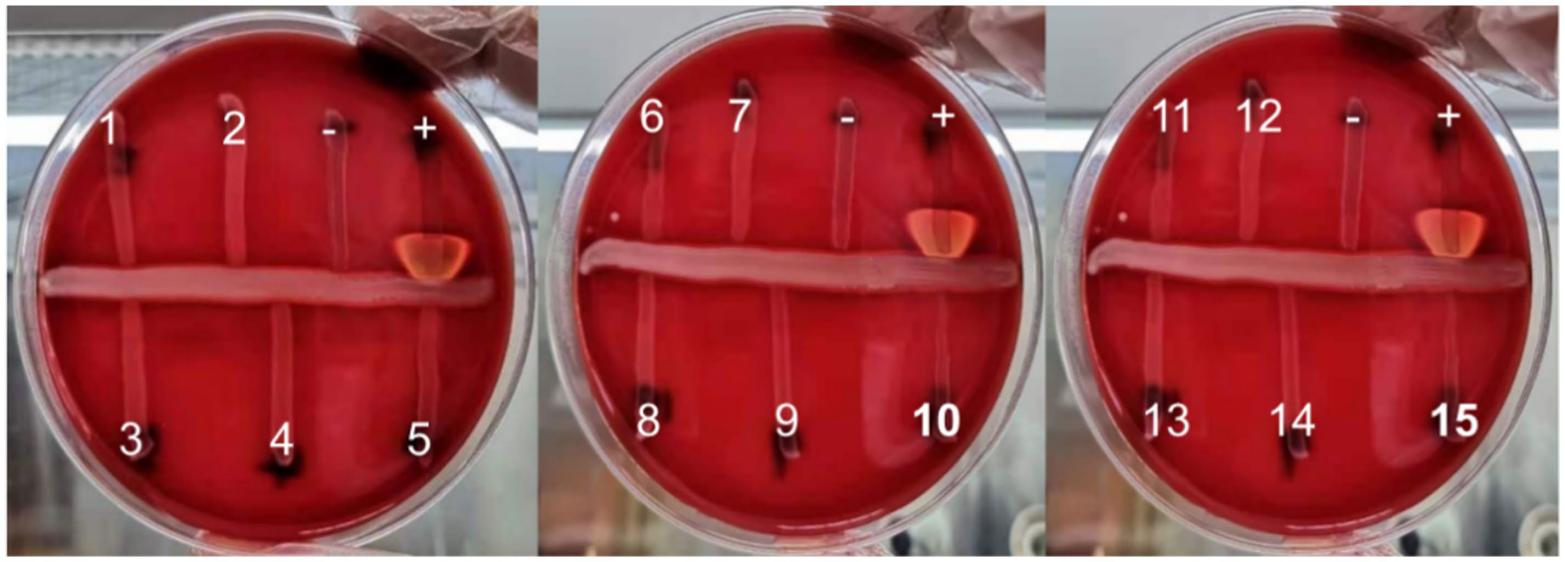
The CAMP test of 15 suspected CAMP-negative isolates of *Streptococcus agalactiae* (+: positive control, −: negative control).

**Figure 2:**
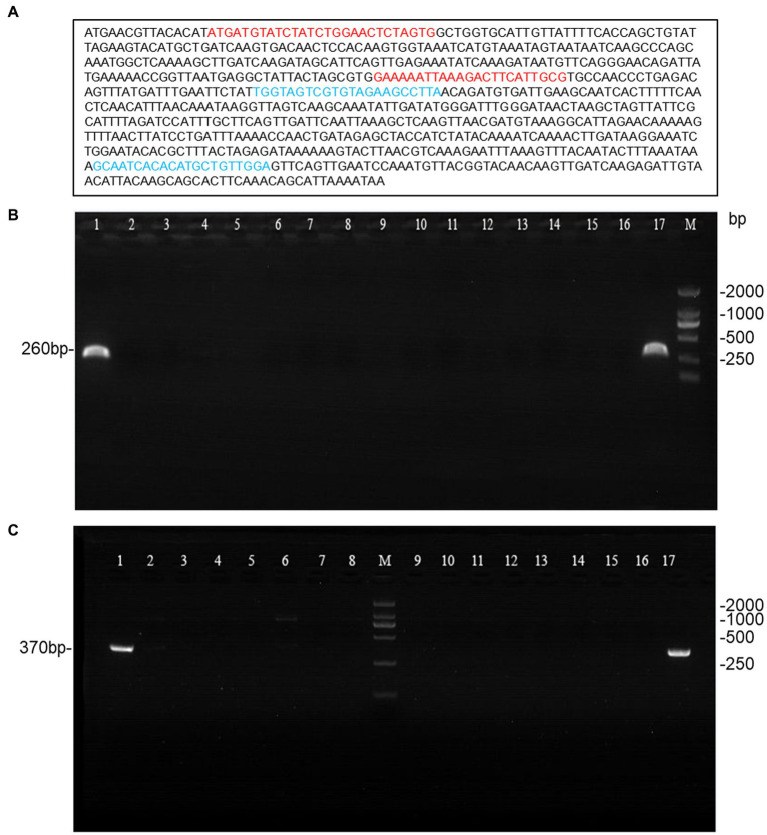
Agarose gel electrophoresis of the amplified *cfb* gene from 15 CAMP-negative isolates. **(A)** Primer design for the *cfb* gene, with red primers targeting the upstream region and blue primers targeting the downstream region. **(B,C)** The *cfb* gene amplified with the two sets primers (lane1 and 17 were positive control; lane2-16 were 15 CAMP-negative isolates; M: DL2000 DNA Marker).

A total of 190 *Streptococcus agalactiae* strains were identified using the VITEK-2 and MALDI-TOF MS systems. Among these, 15 isolates were found to be CAMP-negative strains (Figure 1), representing 7.9% of the total strains. Further confirmation of these strains as *Streptococcus agalactiae* was achieved through 16S rDNA sequence blast (data uploaded to NCBI GenBank: OQ680135, OQ680134, OQ680136, OQ680133, OQ680132, OQ680130, OQ680128, OQ680127, OQ680129, OQ680125, OQ680126, OQ680014, OQ680017, OQ680013, OQ680016). The 15 strains were then subjected to MLST typing, revealing that all of them belonged to ST862 (*adhP* = 16, *pheS* = 1, *atr* = 4, *glnA* = 70, *sdhA* = 9, *glcK* = 3, *tkt* = 2). The *cfb* gene was amplified using two sets of primers, one targeting the upstream region (Figure 2A, Red) and the other targeting the downstream region (Figure 2A, Blue), Agarose gel electrophoresis results indicated chromosomal deletions of the *cfb* gene in these 15 strains (Figures 2B,C), the sequence of positive control for the *cfb* gene was uploaded to NCBI GenBank: OQ871565 and OQ693682.

### 3.2. Antibiotic susceptibility

No GBS resistance to penicillin, ampicillin, vancomycin and linezolid was detected. The resistance rates of the 190 GBS strains to erythromycin, clindamycin, tetracycline and levofloxacin were 62.63, 56.32, 82.11 and 16.84%, respectively. Among these, the resistance rates of CAMP-positive GBS to erythromycin, clindamycin, tetracycline and levofloxacin were 63.43, 56.57, 85.14 and 18.29%, respectively. In contrast, the resistance rates of CAMP-negative GBS were 53.33, 53.33, 46.67 and 0% for erythromycin, clindamycin, tetracycline, and levofloxacin, respectively. No significant difference was observed in resistance rates for erythromycin, clindamycin and levofloxacin; however, a statistically significant difference was found for tetracycline resistance (*p* < 0.01). The results are presented in Table 1.

**Table 1:** Comparison of antibiotic resistance rates between CAMP-positive and CAMP-negative GBS strains.

Antibiotic	CAMP-positive GBS		CAMP-negative GBS		*χ2*	*p*-value
	n/Total	Rate (%)	n/Total	Rate (%)		
Penicillin	0/175	0	0/15	0	–	–
Tetracycline	149/175	85.14	7/15	46.67	13.921	0.000
Erythromycin	111/175	63.43	8/15	53.33	0.602	0.438
Clindamycin	99/175	56.57	8/15	53.33	0.059	0.808
Vancomycin	0/175	0	0/15	0	–	–
Ampicillin	0/175	0	0/15	0	–	–
Levofloxacin	32/175	18.29	0/15	0	2.122	0.145
Linezolid	0/175	0	0/15	0	–	–

n, number of antibiotic resistant strains.

## 4. Discussion

GBS can cause miscarriage, premature delivery, and premature rupture of membranes through ascending infections in the birth canal of pregnant women. It can also lead to neonatal sepsis and meningitis through vertical transmission between mother and child. Consequently, GBS is a pathogenic bacterium that requires close monitoring during the perinatal period (Simonsen et al., 2014).

Christie et al. first reported in 1944 that the CAMP factor exhibits high specificity for GBS (Christie et al., 1944). In 1979, Bernheimer et al. isolated and purified the CAMP factor (Bernheimer et al., 1979), and its coding gene, *cfb*, was discovered in 1994 (Podbielski et al., 1994). Subsequent studies revealed that almost all GBS strains contain the *cfb* gene encoding the CAMP factor. However, some research reports identified CAMP-negative phenotypes (Hassan et al., 2002). The CAMP-negative phenotype in GBS strains with the *cfb* gene may result from transcription defects, low gene expression, or low CAMP factor activity (Podbielski et al., 1994). Between 2012 and 2018, Tickler et al. collected 31 GBS strains from 12 laboratories in the United States and Ireland, which contained deletions in or near the chromosomal region encoding the hemolysin gene *cfb*, but only 5 strains lacked the complete *cfb* gene (Tickler et al., 2019). CAMP-negative GBS has also been identified in China, Guo et al. isolated 4 CAMP-negative strains from 22 GBS strains, but only 1 strain had a *cfb* gene deficiency (Guo et al., 2019). In this study, we used two sets of primers targeting the upstream and downstream regions of the *cfb* gene and ultimately identified 15 *cfb*-deficient GBS strains from 190 isolated GBS strains, with a deficiency rate of 7.9% (15/190). This finding contrasts with the current understanding that the vast majority of GBS (>98%) contain the *cfb* gene and express the CAMP factor (Jorgensen et al., 2015).

Since most GBS strains contain the *cfb* gene, many laboratories and companies use the *cfb* gene as a target for primer design and PCR detection of GBS in vaginal/rectal swabs from pregnant women during the perinatal period (Goudarzi et al., 2015; Tanaka et al., 2016; Ferreira et al., 2018). However, our results indicate that using a GBS detection kit designed for the *cfb* gene in this region may lead to missed detections. The recently developed Xpert GBS LB XC test targets two unique GBS genes: glucosyl transferase family gene and *LysR* family gene, exhibiting higher sensitivity and specificity compared to traditional methods (Thwe et al., 2022).

The MLST results of the 15 CAMP-negative strains showed that they all belonged to the ST862 type. This type has been previously reported by Cheng et al. in Guangzhou, South China. However, the six ST862 GBS strains they identified all carried the *cfb* gene (Cheng et al., 2020), contrasting with our findings. We speculate that this discrepancy may be due to local clonal expansion. The reason for the loss of the *cfb* gene requires further investigation.

Although the resistance rates of CAMP-negative GBS and CAMP-positive GBS to erythromycin and clindamycin were not statistically significant, the resistance rate of CAMP-negative GBS to tetracycline was much lower than that of CAMP-positive GBS. Whether the absence of the *cfb* gene affects GBS drug resistance warrants further study.

The CAMP factor is a secreted protein with perforating properties, known to weaken the host’s immune function during systemic infection (Kurosawa et al., 2016). *In vivo* experiments have demonstrated that the CAMP factor can contribute to, or even cause, animal death, leading to the belief that it is an essential pathogenic factor (Rajagopal, 2009). However, Hensler et al. (2008) conducted *in vitro* and *in vivo* experimental studies after allelic replacement of the *cfb* gene and concluded that the CAMP factor is not necessary for GBS virulence. Therefore, there are still controversies regarding the role and mechanism of the CAMP factor in the infection process. The pathogenicity of these *cfb*-deficient GBS strains isolated in this study requires further investigation.

## 5. Conclusion

Based on the results of this study, 7.9% of GBS isolated from the vagina/rectum of pregnant women were CAMP-negative. As a result, the CAMP test should not be solely relied upon as a presumptive method for GBS identification. Utilizing primers targeting the *cfb* gene could lead to missed detections of GBS, and thus, alternative or multitarget approaches are warranted to ensure optimal diagnostic accuracy.

## Data availability statement

The datasets presented in this study can be found in online repositories. The names of the repository/repositories and accession number(s) can be found below: NCBI - OQ680135, OQ680134, OQ680136, OQ680133, OQ680132, OQ680130, OQ680128, OQ680127, OQ680129, OQ680125, OQ680126, OQ680014, OQ680017, OQ680013 and OQ680016.

## Ethics statement

The studies involving human participants were reviewed and approved by the Ethics Committee of Pingshan District People’s Hospital of Shenzhen. The patients/participants provided their written informed consent to participate in this study.

## Author contributions

JZ, TF, and SY designed the experiments. JZ, LZ, HL, KX, YZ, and BZ executed the experiments. JZ, LZ, TF, and SY conducted the data analysis. KX and BZ collected/provided the clinical samples and information. JZ and SY wrote the manuscript with input from all of the other authors. All authors contributed to the article and approved the submitted version.

## Funding

This study was supported by grants from the National Natural Science Foundation of China (No. 82272986 to SY), the Natural Science Foundation of Guangdong Province, China (No. 2023A1515010230 to SY), the Science and Technology Foundation of Shenzhen (No. JCYJ20220531094805012 to SY), the Project of Educational Commission of the Guangdong Province of China (No. 2022KTSCX022 to JZ), and the National Key R&D Program of China (No. 2020YFC2006400 to TF).

## Conflict of interest

The authors declare that the research was conducted in the absence of any commercial or financial relationships that could be construed as a potential conflict of interest.

## Publisher’s note

All claims expressed in this article are solely those of the authors and do not necessarily represent those of their affiliated organizations, or those of the publisher, the editors and the reviewers. Any product that may be evaluated in this article, or claim that may be made by its manufacturer, is not guaranteed or endorsed by the publisher.

## Footnotes

^1^ http://pubmlst.org/sagalactiae/
